# 3D-printing allows for fluid-controlled linear actuators with unconventional shapes

**DOI:** 10.1016/j.heliyon.2024.e26497

**Published:** 2024-02-21

**Authors:** Eva Zillen, Bob van der Windt, Heike Vallery, Gerwin Smit

**Affiliations:** a Delft University of Technology, Department of Biomechanical Engineering, Faculty of Mechanical Engineering, the Netherlands; b RWTH Aachen, Department of Automated Control, Faculty of Mechanical Engineering, Germany

**Keywords:** 3D-printing, Additive manufacturing, Pneumatic actuators, Hydraulic actuators, Gas springs, Piston-cylinder systems, Shape-independent pneumatic actuators

## Abstract

**Background:**

Pneumatic actuators are widely used in applications like (medical) robots, or prostheses. Pneumatic actuators require a complex manufacturing process and are produced in standardized dimensions to reduce costs. Over the last decade 3D-printing has emerged as a cost-effective and efficient production method in medical applications. 3D-printing can also function as a cost-efficient alternative production method for pneumatic actuators.

**Objective:**

The goal of this research is to study the possibility of creating a pneumatic linear actuator with 3D-printing. Furthermore, the aim is to use the advantage of 3D-printing to create pneumatic actuators with non-circular cross-sections.

**Methodology:**

To evaluate the performance of a 3D-printed pneumatic actuator, a test setup was designed and built to measure the leakage and sliding friction force. Furthermore, two pneumatic actuators with a non-conventional cross-sectional shape were designed and their performance was tested and compared with a 3D-printed cylindrical pneumatic actuator, since these tests only ran once, the results are more a guideline. During the manufacturing of the cylinders, no post-processing techniques were used.

**Results:**

The functioning of a 3D-printed circular pneumatic actuator was proven with low static leakage rates of 2.5%, low dynamic leakage rates of approximately 1%, and a maximum friction force of . Furthermore, the results show that it is possible to print functioning pneumatic cylinders with a non-cylindrical concave cross-section. The non-conventional cylinders were tested up to  with maximum dynamic leakage of .

**Conclusion:**

This study demonstrates a method to create functional pneumatic linear actuators with 3D-printing. It was possible to create 3D-printed actuators with a conventional shape, e.g. circular and unconventional shapes e.g. stadium/oval shape and a kidney shape. The leak rates for conventional and unconventional shapes were in the same range. This opens up the world for more design freedom in pneumatic actuators.

## 1 Introduction

### 1.1 Motivation

Pneumatic actuators and gas springs are widely used in the field of prosthetics and orthotics [1]. These actuators can restore a large amount of energy, are lightweight, and have a compact volumetric profile [2]. However, pneumatic actuators require a complex manufacturing process [3] and to reduce manufacturing costs most pneumatic cylinders are produced in standardized dimensions, making it hard to customize the actuators to different users [4]. At last the implementation of pneumatic cylinders lacks design freedom during system integration due to their cylindrical piston shape. A possible solution for the manufacturing of pneumatic actuators is 3D-printing. 3D-printing allows for high design freedom since complex parts can be designed and created that are practically impossible to manufacture conventionally, e.g. parts with internal passageways [5]. Furthermore, 3D-printing gives the ability to easily create low-cost customer-specific products. At last, 3D-printing has low production costs, as specialized and expensive manufacturing equipment is not necessary. 3D-printing is used in various industries such as robotics, automotive, and aerospace industries [6]. Besides these industries, the use of 3D-printing in the medical sector is also rapidly growing. 3D-printing is used to create medical tools, an overview can be found in the study of Culmone et al. [7], medical implants or devices like exoskeletons, prostheses, and orthoses [8].

Implementing 3D-printing in gas spring manufacturing also has advantages in piston shapes. The design freedom of 3D-printing theoretically allows for creating non-circular piston shapes. The use of non-circular piston shapes can be very beneficial in prosthesis and orthosis design. For example a very important design constraint for prosthesis and orthosis design is that a prosthesis or orthosis must fit inside someone's pants. Especially with the use of a circular pneumatic cylinder this will be hard, due to the diameter of the piston, but what if a flat oval actuator could be implemented? This will make it possible to fit an orthosis inside the design envelope.

**Figure 1:**
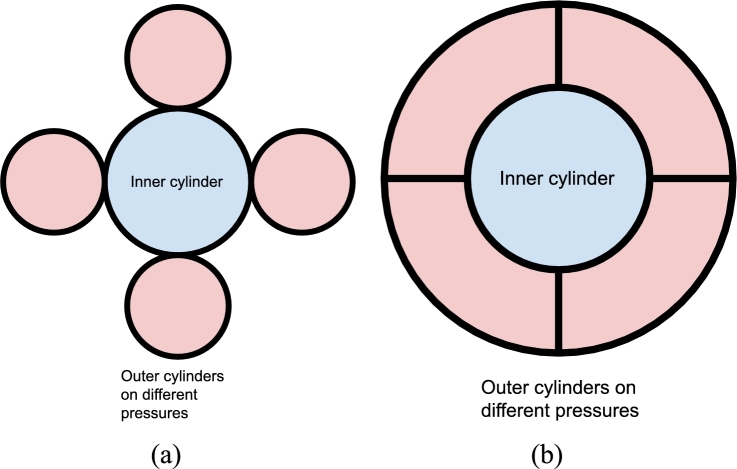
Schematic top views of two multiple cylinder systems. It can be seen that with an integrated system (Fig b) the surface area of the outer cylinders is larger compared to the non-integrated cylinder system (Fig a).

Another option is to integrate multiple actuators in a smaller building environment. This can be useful in prosthesis research where pneumatic actuators with different stiffnesses are needed. To fit multiple actuators in one building envelope the use of non-circular cylinders can be helpful. Fig. 1 shows a schematic top view of a cylindrical and non-cylindrical stack of multiple cylinders.

### 1.2 State of the art — 3D-printed pneumatic actuators

Previous attempts at creating 3D-printed pneumatic actuators have mostly focused on flexible pneumatic actuators to create grippers [9], [10], or for rehabilitation devices [11]. Also in linear artificial muscles some efforts are made in using 3D-printing for manufacturing [12], [13]. Some studies describe the design of a 3D-printed non-flexible linear pneumatic actuator. Krause and Bhounsule created a double-acting pneumatic piston-cylinder system of mostly 3D-printed parts [14]. The parts were printed with a fused deposition modeling (FDM) printer using PLA material, some parts were reinforced to prevent failure. After post-processing steps, it showed comparable strength and performance to commercially available actuators. A miniature double-acting 3D-printed pneumatic actuator was designed by Nall and Bhounsule [15]. This double-acting actuator was printed with an FDM printer using ABS as material. Just like in the study performed by Krause and Bhounsule, the piston was reinforced with a steel part. After the inner cylinder wall was post-processed with chemicals to create a smooth surface finish, the actuator showed a comparable power-to-weight ratio to commercial actuators of the same size. Similar work to this study is the thesis of Martinez de Apellaniz Goenaga [16], who printed single-acting hydraulic cylinders with different printing techniques — FDM, Stereolithography (SLA), and selective laser melting (SLM). De Apellaniz Goenaga et al. found that SLA printing gave the best results, and processing the cylinder wall with a reamer lowered the friction force in the system. Besides 3D-printing, another fast prototyping option for the manufacturing of pneumatic actuators was studied by Groenehuis and Stramigioli [17]. They designed non-conventional pneumatic actuators that could be produced with laser-cutting techniques. No published papers were found on 3D-printed pneumatic gas springs or actuators where no post-processing steps were needed to prevent leakage. Also, no previous work on 3D-printing pneumatic actuators with a non-conventional cross-sectional piston shape was found.

Important work in the field of comparing different commercially available cylinders is done by Tran and Yanada [18]. They present a method to determine the friction in a pneumatic cylinder and compare commercially available cylinders.

### 1.3 Friction force in pneumatic actuators

When designing a pneumatic actuator, one of the most important aspects is to prevent leakage. Applying a seal is essential to prevent leakage in a pneumatic actuator, but at the same time highly increases the friction within the system. Friction is always present in a pneumatic cylinder system [18], but the magnitude of the friction force depends on several aspects, which will be further discussed in subsection 3.1.3. When designing the 3D-printed pneumatic actuator, the aim is to prevent leakage while keeping the friction forces to a minimum.

### 1.4 Objectives

This paper aims to investigate the performance of a 3D-printed pneumatic actuator, without post-processing steps. For the sealing of the piston, a commercially available sealing will be used. To analyze the performance of a 3D-printed piston-cylinder system, the focus is to prevent leakage and at the same time maintain a low friction force. To study the full potential of 3D-printed pneumatic actuators the research is split into two subgoals, first, the leakage and friction behavior of a 3D-printed pneumatic actuator with a cylindrical piston and standardized sealing are measured and analyzed. Secondly, two non-circular pneumatic actuators are designed and their performance is measured and compared with the performance of the 3D-printed circular pneumatic actuator.

## 2 Design and manufacturing of 3D-printed actuators

### 2.1 Circular actuators

#### 2.1.1 Pneumatic actuator design

All designed pneumatic actuators in this study are single-acting cylinders that can act as air springs. The clearance between the piston and the cylinder wall in off-the-shelf actuators is usually between 50 and  [19]. However, because 3D-printing with an SLA-printer (see 2.3) has a limited surface finish, a higher clearance of  () was selected to prevent friction between the sliding interface of the piston and the cylinder wall. All cylinders are designed to have a stroke length of . Table 1 shows the diameter of the circular actuator.

**Table 1:** Dimensions of the piston shapes.

Shape	Dimensions O-ring	Defined variable		
Circular shape		d		
			

Stadium shape		*L*	*D*	
			

Kidney shape		*r*	*a*	*γ*
				$\frac{5}{9}\pi$ rad ()

#### 2.1.2 Sealing selection

The O-ring, a soft rubber ring, is a standardized sealing and therefore the feasibility of the 3D-printed pneumatic actuators was tested with this sealing type. A double-acting Parkside O-ring with a  outer diameter was used. Another option is to 3D-print sealings, especially for the unconventionally shaped pistons this is a good solution. The sealings can be printed in a different printer setting with a different material or with a multi-material printer. The authors decided to use standardized O-rings because the focus is on varying the shapes of pneumatic actuators with 3D-printing, with the 3D-printing sealings an extra variable is introduced. Furthermore, the sealing is a part that can be damaged easily, with a commercial O-ring it is simple to replace such a part.

#### 2.1.3 Piston grooves

The groove for the O-ring was designed to maintain a  squeeze ratio when placed in the cylinder, this is in line with the Eriks O-ring installation guide [20].

### 2.2 Non-circular actuators

**Figure 2:**
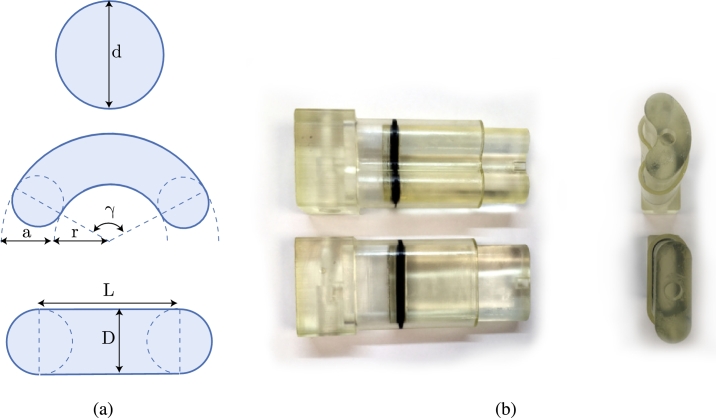
(a) The piston shape of the tested actuators: A circular piston shape with diameter *D*, a kidney shape with variables *a*, *r*, and *γ* and a stadium shape with variables *L* and *D* (b) 3D-printed and assembled prototypes of the stadium shape and kidney shape pneumatic actuators.

Two pneumatic actuators with a non-circular cross-section were designed. It is not possible to manufacture these actuators using conventional methods [5]. An O-ring was used as a sealing mechanism because this sealing can easily be shaped to the non-circular piston. The first additional piston shape is a stadium shape – a rectangle with semicircles at opposite ends. The second piston shape resembles a kidney. Fig. 2(a) shows both non-circular piston shapes. The stadium- and kidney-shaped actuators were tested with the same test setup as the circular cylinders. In order to fairly compare the designed pneumatic actuators, the non-conventional shaped cylinders were designed to have an equal cross-sectional surface area to the circular cylinder. At the same time, it was ensured that the inner perimeter of the cylinder was equal to the outer perimeter of the O-ring for proper sealing. The used O-ring has an internal diameter of  and a cross-sectional diameter of . Table 1 and Fig. 2(a) give an overview of the dimensions used. All non-circular actuators are designed to have the same stroke length as the circular actuators, . Fig. 2(b) shows the printed prototypes.

### 2.3 Printing method

All designed actuators were printed with a Formlabs 3 SLA printer. In preliminary research done by the authors, the static leakage of an air chamber printed on an FDM printer (Ultimaker S5), a formlabs 3 and a machined air chamber of aluminum were tested. It was found that air chambers printed with a Formlabs 3 printer had the lowest static leakage of the tested printing techniques and showed comparable results with the machined air chamber [21]. Martinez de Apellaniz Goenaga also shows low friction force in 3D-printed hydraulic cylinders, printed with an SLA-printer [16]. The material used for all designs is a Formlabs material: *ClearV4* [22]. A layer height of  was selected to save printing time. Orientation is important as well for 3D-printing leakage-free components. Since this research focuses on applying unconventional shapes there is no study done on the optimal orientation, an upright position for all cylinders and pistons was chosen.

## 3 Experimental evaluation

### 3.1 Experimental setup

#### 3.1.1 Static leakage tests

**Figure 3:**
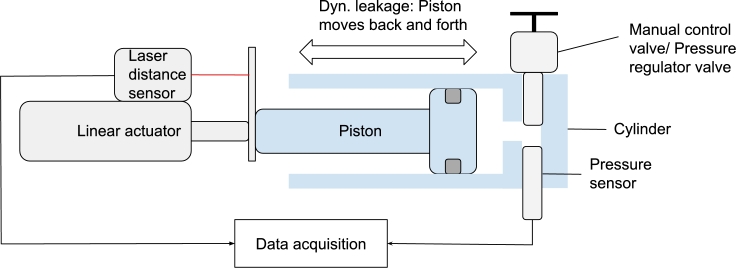
Schematic overview of the test setup, in the static leakage test the cylinder moved to a high pressure position, in the dynamic leakage and friction range test the cylinder moved back and forth. In the friction range test, the manual control valve was replaced with a pressure regulator and a compressed air supply.

Fig. 3 shows a schematic overview of the test setup to measure leakage in the pneumatic actuators. For static leakage, the piston was set to a fixed high-pressure position of  and stood still at this position for the complete test. The pressure was measured for 20 minutes. The pressure drop during the tests was interpreted as static leakage.

#### 3.1.2 Dynamic leakage tests

To measure the dynamic leakage within the system, the same test setup was used as for the static leakage test, see Fig. 3. This test was designed to measure the dynamic leakage when the piston moves inside the cylinder. The piston was moved back and forth over the complete stroke for 20 minutes, around 200 times. The pressure difference at a predetermined position in the cylinder was interpreted as the dynamic leakage of the sealing.

#### 3.1.3 Friction force tests

The third test in this study was performed to determine the friction force generated over the stroke of the pneumatic actuator. Non-linear behavior makes modeling the dynamic friction force in a fluid-controlled actuator a complex task. From previous work, it is known that the friction force is dependent on both the velocity in which the piston moves and the pressure on which it operates [18], [23], [24], [25]. Furthermore, specific properties of the actuator have an influence on the frictional force. According to Wassink et al., lip seal friction under constant speed sliding can be modeled as the sum of three physical components [24]:

• Viscous shear loss in the lubricant.
• Hysteresis losses due to roughness-imposed deformation of the seal material.
• Hysteresis losses due to deformation caused by varying intermolecular forces at the sliding interface.

In this study, the same lubricant was used for each cylinder — Rocol Kilopoise 0001. Fig. 3 shows a schematic overview of the friction test. The piston was moved inside the cylinder and during the tests, the pressure was kept at a constant level with a pressure regulator. During the tests, the force was measured on the piston.

**Figure 4:**
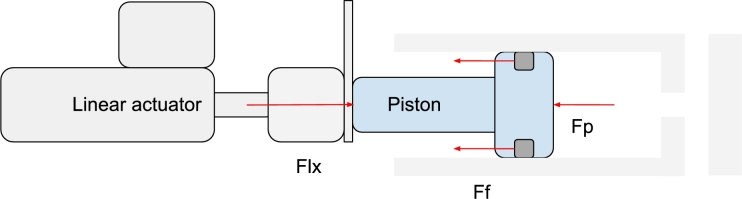
A simplified section view diagram of the piston in the friction test. Forces in the y-, and z-direction are excluded for simplicity reasons.

A simplified diagram of the piston in this test is shown in Fig. 4. Forces in the y-, and z-direction are excluded for simplicity reasons. To find the friction force, $F_{F}$, the forces in the x-direction were analyzed in equation (1),

(1) $$F_{LX}-F_{F}-F_{p}=m\frac{dv}{dt}.$$

$F_{LX}$ is the force exerted by the load cell, $F_{p}$ is the resultant force exerted by the gauge pressure, *m* is the mass of the piston, and *v* is the velocity of the piston. To calculate the friction force, it is assumed that the piston velocity is constant and therefore the system is interpreted as a steady-state system. The force $F_{p}$ is calculated in equation (2) by multiplying the measured pressure (*p*) with the surface area of the tested cylinder (*A*):

(2) $$F_{p}=p\cdot A.$$

The friction force is then calculated in Equation (3):

(3) $$F_{F}=F_{LX}-F_{p}.$$

#### 3.1.4 Repeatability of the circular pneumatic actuator

The static leakage test, dynamic leakage test, and friction range test were repeated for the cylindrical actuator. The tests ran three times in a row, without changing the connections of the air inlet and pressure sensor. It is important to note that the tests with the non-cylindrical pistons were not repeated as these tests were only used as proof of concept of actuators with unconventional piston shapes.

#### 3.1.5 Reducing the clearance in non-circular actuators

Additional tests were done with two non-circular cylinders with smaller clearance. For the two non-circular piston shapes, the clearance was reduced from  () to  (). The same method was used to perform the static leakage, dynamic leakage, and friction tests as described in Section 3.1.1, 3.1.2, 3.1.3.

#### 3.1.6 Test setup design

**Figure 5:**
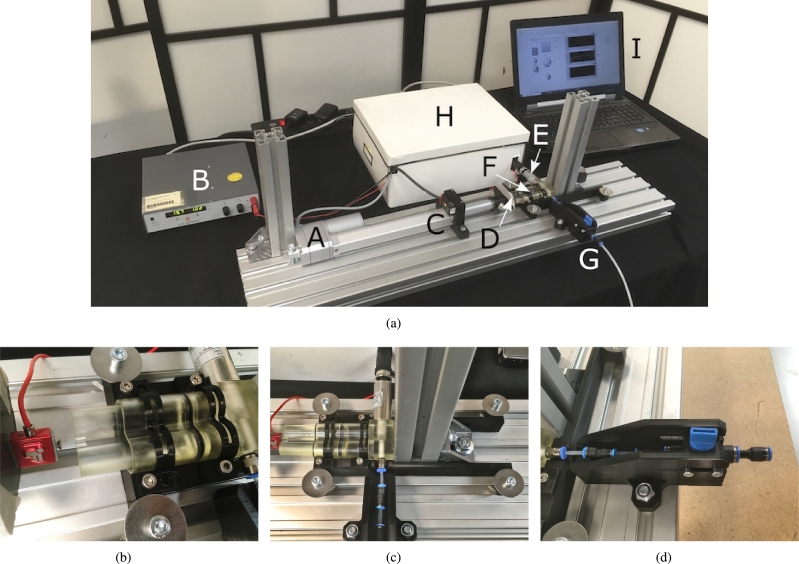
A picture of the experimental test setup consisting of an linear actuator (A) with its power source (B), a laser distance sensor (C), a force sensor (D), a pressure sensor (E), the 3D-printed test model (F), an air inlet with manual control valve (G), a box with electrical components (H) connecting the sensors via LabVIEW to a computer (I).

Fig. 5 shows the designed test rig. During the tests, high forces up to  are measured at pressures around . To prevent deflexion in the system, an aluminum frame is used as a base to connect all different elements. An linear actuator (A, DSZY1-potentiometer), with its power source (B), is connected to the base. The laser distance sensor (C, Micro Epsilon optoNCDT) is connected to the other side of the linear actuator. The force sensor (D, Futek Miniature S-Beam Jr. Load Cell 2.0) is placed at the end of the electrical piston. The side of the force sensor which is connected to the piston of the tested cylinder has a protruding element, which ensures the alignment of the piston (see Fig. 5(b)). The force sensor pushes the piston of the 3D-printed cylinder (F). A part was designed to align the test element during the tests, see Fig. 5(c). The pressure sensor (E, SensorTechnics CTU8000), measures the pressure during the tests. The manual control valve (G, Festo Shut-off valve) is fixated on the base with a connecting part (Fig. 5(d)), to prevent movement of the connecting elements during the test. The sensors are connected via LabVIEW to a computer (I). To counteract the high axial forces and prevent undesired tensions in the system, both sides are strengthened by aluminum pillars which can be seen in Fig. 5(a).

### 3.2 Data analysis

#### 3.2.1 Data analysis of static leakage results

To adequately visualize the results of the static leakage test, the data required post-processing. A sliding averaging window of value 13, Matlab command *movmean()*, and sampling of the data with  in Labview were applied.

#### 3.2.2 Data analysis of dynamic leakage results

Ideally, the dynamic leakage is measured as a difference between the pressure peaks at the start and at the end of the dynamic cycle. However, due to an overshoot of the electric linear actuator, this is not possible. The overshoot of the electric cylinder is caused by the force-dependent control loop of the electric cylinder causing overshoots at the end of the stroke of the tested pneumatic actuator. To analyze the dynamic leakage, the pressure at a specific distance in the stroke of the cylinder was found and checked over time. In order to find this pressure, the data from the distance sensor was combined with the data from the pressure sensor. A variable *α* was defined as the position in the cylinder where the pressure was analyzed. This variable slightly differs per test model, to ensure equal starting pressure for every model. The laser distance sensor provides accurate values (in ) up to three decimals. For the analysis of the dynamic leakage, all pressures were selected in a range of *α* to *α*+ of the laser distance sensor data. Table 2 shows the values of *α*.

**Table 2:** Cylinder position, *α* for each piston shape.

Test model	*α*
Circle	
Stadium	
Kidney	

#### 3.2.3 Data analysis of friction force range

**Figure 6:**
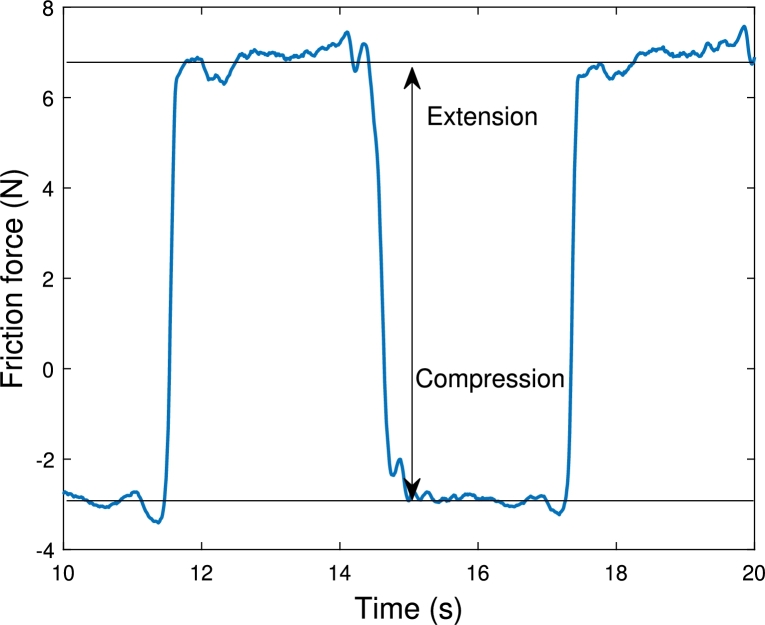
The friction force range definition at . The arrow indicates the difference between the mean friction force in the extension and compression stroke of the cylinder.

Fig. 6 shows a visualization of the friction force at . The difference was taken between the friction force of the extending and compression stroke. This metric is called the friction force range. For each of the ten movement cycles, the friction force range is determined.

## 4 Results

### 4.1 Cylindrical pneumatic actuator

#### 4.1.1 Static leakage

**Figure 7:**
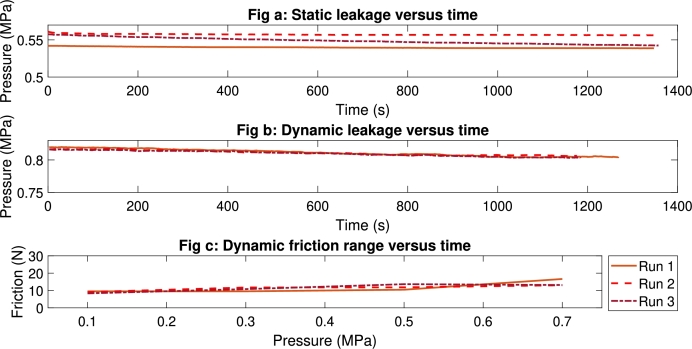
Repetability tests for a 3D-printed circular pneumatic actuator. Fig(a) shows a static leakage test for 3 runs, Fig(b) shows a dynamic leakage test for 3 runs, and Fig(c) shows a dynamic friction range test for 3 runs.

Fig. 7a shows the static leakage results of the cylindrical piston. Fig. 7a shows a maximum static leakage of , or  of the starting pressure. The first test started at a lower pressure of approximately  instead of .

#### 4.1.2 Dynamic leakage

Fig. 7b shows the results of the dynamic leakage tests. Fig. 7b shows minimal differences between the test data and a maximal dynamic leakage of  or .

#### 4.1.3 Friction force

Fig. 7c shows comparable results for the three tests. At  run 1 shows higher friction forces than run 2 and 3, a friction force of  compared to .

### 4.2 Non-circular actuators (stadium and kidney shape)

#### 4.2.1 Static leakage

**Figure 8:**
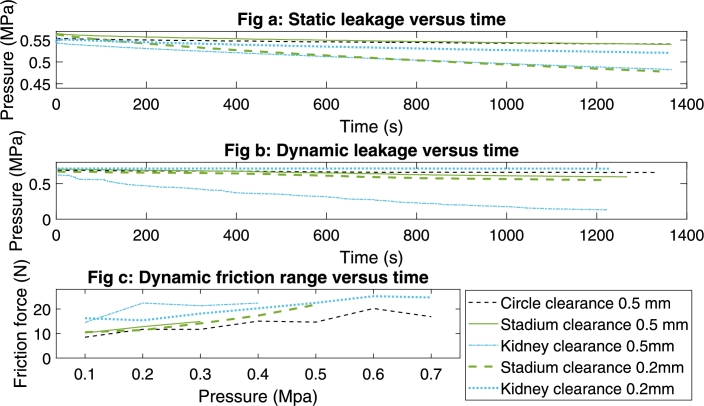
Results of the static leakage test (Fig(a)), the dynamic leakage test (Fig(b)) and the dynamic friction range test (Fig(c)) for three different piston shapes and two different clearances. In Fig(a) and Fig(b) the leak rates of the stadium shape with 0.2 mm are higher due to damage to the connector threads.

The results of the static leakage test of the non-circular actuators are illustrated in Fig. 8a. The cylindrical piston shape performs best in the static test with a pressure drop of approximately . This is approximately 1.8% pressure loss. The stadium-shaped model lost double the amount of pressure  over the duration of the test. This is a pressure loss of approximately 3.6%. The kidney-shaped model dropped  of pressure during the test, which is approximately 11% pressure loss.

#### 4.2.2 Dynamic leakage

To ensure a valid comparison of the non-cylindrical models to the cylindrical model, values of *α* were hand-picked. The position in the cylinder was selected at an equal starting pressure for all three models. Table 2 shows the values of *α* which were used in the data analysis. Fig. 8b shows the results of the dynamic leakage of the non-circular actuators. The pressure in the circular piston shape remains most constant over time. The stadium piston shape showed a leakage of approximately , , after 200 cycles. The kidney piston shape actuator produced a high dynamic leakage, as during the test the pressure in the cylinder decreased from  to merely , as can be seen in Fig. 8b.

#### 4.2.3 Friction force

**Figure 9:**
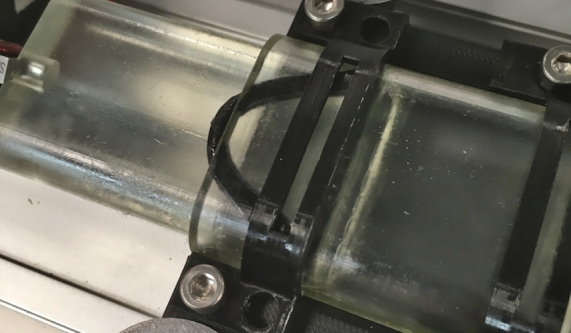
The O-ring moves out of the clearance gap at pressures from  in the stadium shaped piston.

During the friction force tests of the varying cross-sectional-shaped cylinders, the placement of the O-ring in the piston groove was less stable in the non-circular shapes compared to the circular model. When increasing to higher pressures, the O-ring moved out of the clearance gap. Fig. 9 shows the O-ring moving out of the clearance between the piston and the cylinder. For this reason, the friction force was measured at increments of  instead of the pre-defined pressure values. Interestingly, the kidney-shaped model could withstand a higher pressure compared to the stadium model before the movement of the O-ring. The stadium-shaped and the kidney-shaped models were tested up to a pressure of  and  respectively. Fig. 8c shows that the kidney-shaped actuator has the highest friction force range. Compared to the other models, the kidney shape showed more deviation between the different pressure levels. The friction force range was  at ,  at ,  at , and  at . The friction force of the stadium piston shape is comparable to the circular-shaped actuator.

#### 4.2.4 Reducing the clearance in non-circular actuators

Figs. 8a and b show the static leakage and dynamic leakage results with lower clearance. The stadium piston shape showed more leakage in this follow-up test. A possible explanation is damage to the hose connector threads. However, when looking at the low-clearance kidney piston shape, expectations are exceeded. There are improvements for all three tests performed on this model. The results for the dynamic leakage test are especially promising, where the leakage was reduced to almost zero over the 200 cycles. The low leakage rates confirm the feasibility of 3D-printing differently shaped actuators and at the same time show its huge potential.

Fig. 8c shows the results of the follow-up friction tests performed with these models. The friction results showed two advantages of lowering the clearance (Fig. 8c). Firstly, higher pressure levels could be reached before the O-ring was moved out of the clearance, as shown in Fig. 9. Secondly, both models showed a lower friction force compared to the corresponding models with a clearance of .

## 5 Discussion

### 5.1 3D-printing pneumatic actuators

This study shows the possibility of producing pneumatic actuators using 3D-printing, SLA-printing, without performing any post-processing steps and evaluates their performance based on leakage and friction. Generally speaking, the results demonstrate that it is possible to build a functional 3D-printed pneumatic actuator. Furthermore, it shows the possibility to create custom 3D-printed pneumatic actuators with various piston shapes which opens the door to more design freedom.

### 5.2 Repeatability of circular actuator

#### 5.2.1 Static leakage

Although run 1 started at a lower pressure level in the repeatability tests the leakage results follow the same profile. Only run 3 in Fig. 7a shows higher leakage of  or . A possible explanation for the higher leakage rate is resettling or rotation of the O-ring due to a higher clearance.

#### 5.2.2 Dynamic leakage

Fig. 7b shows that the dynamic leakage results have a small variance and follow the same curve.

#### 5.2.3 Friction range

The friction ranges in Fig. 7c show comparable results. The variation of friction forces in different runs can be explained by breakaway friction and the redistribution of grease [26].

### 5.3 Non-circular piston shapes

It is important to stress that the tests for non-circular pistons only ran once. Therefore the results are more of a guideline for future investigation into leakage in non-circular pneumatic actuators.

#### 5.3.1 Stadium piston shape

Compared to the circular-shaped actuator, the stadium-shaped model only showed slightly larger pressure losses. This is mainly due to the movement possibilities of the O-ring in the piston groove. On the flat side of the stadium piston, the pretension of the circular O-ring is lower, resulting in a larger gap between the piston groove and the O-ring and more movement possibilities. Furthermore, the actuator was designed with a higher clearance compared to conventionally manufactured actuators as described in section 2, giving more clearance for the O-ring to move or rotate in its groove. The high pressure in the cylinder during the friction test caused a shear stress in the cylinder wall, which created a small deformation of the cylinder wall. This further increased the possibility for the O-ring to move out of the clearance. Therefore the test could be performed up to . Fig. 8c shows that the friction force of the stadium piston shape is slightly higher with all pressure levels compared to the circular piston shape. The higher friction force is a result of the larger perimeter of this piston shape, leading to an increase in friction.

#### 5.3.2 Kidney piston shape

One of the main difficulties of the kidney piston shape is that the used O-ring is circular and will not naturally follow the piston groove. The form-closure of the cylinder wall is important to bring the O-ring into pretension. During the dynamic leakage test and the friction test, rotation of the O-ring was observed. Firstly, Fig. 8b shows a large pressure drop between 300 and 350 seconds, which can be explained by the rotation of the O-ring observed during that time interval. Secondly, Fig. 8c shows in the dynamic friction force range an outlier at a pressure of . During this run, O-ring rotation was observed.

#### 5.3.3 Reducing the clearance

As discussed in the previous subsections, the main problem of actuators with non-conventional piston shapes is the rotation and linear movement of the O-ring in or out of the groove. Two possible improvements could solve this. The first potential improvement is using an X-ring instead of an O-ring because these rings are suitable to prevent rotation of the sealing [27]. The second is by lowering the clearance between the piston and the cylinder, which was done in this research. Lowering the clearance showed an improvement in friction in both unconventional shapes and also an improvement in dynamic leakage in the kidney shape. In the stadium shape, no improvements were made in the leakage data, this is due to damage to the connector threads. Since the tests with lower clearance were only done once, these results are more of a guideline to more in-depth research on unconventionally shaped actuators.

#### 5.3.4 Design freedom in pneumatic actuator design

The results of Fig. 8 show that with lowering the clearance the leakage results for the different piston shapes are comparable. Therefore 3D-printing can lead to more design freedom in the design of pneumatic actuators.

### 5.4 Comparing to the state of the art

No previous work was found that compared the leakage and dynamic sliding friction force of 3D-printed pneumatic actuators. Tran and Yanada evaluated the dynamic sliding friction force of commercial pneumatic actuators of  bore size [18] at different pressure levels and different velocities. The piston velocity of the 3D-printed actuator during one of the runs was calculated. At a pressure of  the velocity of the extending stroke was  and the velocity of the compression stroke was . Table 3 shows the corresponding results of Tran and Yanada for these velocities and pressure levels. The 3D-printed actuator generates a friction force of the same order of magnitude as the commercially available actuators based on this comparison. The 3D-printed actuator even shows less friction force, but this is probably due to the difference in actuator design. Tran and Yanada studied a double-acting sealing, which has an extra rod seal, next to the piston seal. The 3D-printed models only contain a piston seal.

**Table 3:** Found friction force compared to commercially available actuators at .

Model	Stroke	Friction force	velocity
3D-printed actuator	Extending		
	Retracting		
	Entire		

Standard actuator [18]	Extending		
	Retracting		
	Entire		

### 5.5 Strengths and limitations

#### 5.5.1 Limitations

In this paper the assumption is made that the pistons of the circular and non-circular actuators move in a steady state and that there are no accelerations during the movement of the piston. However, since the actuators have a short stroke length of about  this assumption and the friction calculations assuming steady state movements can be discussed. To properly evaluate the friction inside short-stroke actuators more research is needed into the accelerations and decelerations at the stroke of a short-stroke actuator.

The force sensor and the piston rod are rigidly coupled leading to a cross-talk of radial forces and moments in the load cell. Since the load cell can only measure forces in the axial direction an error up to  can occur [21]. To further decrease the error in the force measurement, a universal joint could be used to ensure only axial forces are measured, like Belforte used in the described test set-up [23]. The misalignment can also lead to an extra moment on the O-ring of the piston, resulting in extra leakage.

Furthermore, the velocity of the linear actuator used in the tests is force-dependent. This influences the measurements of the friction force which is dependent on the piston velocity. The friction force was measured at relatively small velocities of around , where extra Stribeck friction (friction at low speeds) can be measured next to the Coulomb friction [18]. For future studies, it is recommendable to use a linear actuator with force-independent velocity control, similar to Belforte et al. used [25]. Another option is to use a hydraulic cylinder to move the tested cylinder, as Tran&Yanada [18] and Belforte et al. [23] did in their studies for dynamic sliding friction force in commercial pneumatic actuators.

At last, the non-circular pneumatic actuators were not tested on repeatability and most tests only ran once. Therefore the results of the non-circular actuators are more of a guideline. For future research, it is recommended to do repeatability tests of the non-circular pneumatic actuators to get results on variability and long-term use.

#### 5.5.2 Strengths

This study demonstrates that 3D-printed cylindrical pneumatic actuators can function and shows functional static and dynamic leakages and friction forces that are in the same order as the state of the art.

Furthermore, this paper presents a low-cost and easy-to-manufacture method to evaluate different 3D-printed actuators on static and dynamic leakage, and to evaluate the friction force range with only three low-cost sensors and easy-to-follow data analysis.

At last, this study shows that is possible to change the piston shape of pneumatic actuators and shows the full potential 3D-printed pneumatic actuators have. The 3D-printed pneumatic actuators with unconventional piston shapes show functional static leakage and low friction graphs. After the lowering of the clearance, the dynamic leakages are comparable to the 3D-printed cylindrical actuators. The tested piston shapes have comparable results regarding static and dynamic leakage, creating more design freedom.

## 6 Conclusion

This study presents the design of three different single-acting 3D-printed pneumatic actuators. A cylindrical pneumatic actuator, a pneumatic actuator with the piston shape of an oval/stadium, and an actuator with the piston shape of a kidney. The designs required no post-processing steps on the cylinder wall. A method is presented to evaluate the performance of the models, concerning static leakage, dynamic leakage, and dynamic sliding friction force.

Although creating a complete leakage-free actuator was not achieved yet, it turned out to be possible to 3D-print three functioning cylinders with different piston shapes. Thereby the leak rates of different piston shapes are comparable, for the preliminary single tests as done in this research. A stadium-shaped pneumatic actuator was printed with a static and dynamic leakage comparable to the 3D-printed circular pneumatic actuator. The results of the kidney-shaped model showed a high leakage rate initially, which was solved after reducing the clearance. The kidney-shaped actuator worked up to a pressure of  and with reduced clearance to the maximum tested pressure level of , with maximum dynamic leakage of  after 200 runs. This opens the world to customized pneumatic actuators, enabling geometry improvements in applications such as prostheses or orthoses.

## CRediT authorship contribution statement

**Eva Zillen:** Writing – original draft, Visualization, Validation, Methodology, Investigation, Data curation, Conceptualization. **Bob van der Windt:** Writing – review & editing, Writing – original draft, Visualization, Validation, Formal analysis. **Heike Vallery:** Writing – review & editing, Supervision, Conceptualization. **Gerwin Smit:** Writing – review & editing, Supervision, Conceptualization.

## Declaration of Competing Interest

The authors declare that they have no known competing financial interests or personal relationships that could have appeared to influence the work reported in this paper.

## Data Availability

The full data of the tests performed in this study, together with the python scripts to process the data will be available after publication.
